# Association between neuropathic pain characteristics and DNA methylation of transient receptor potential ankyrin 1 in human peripheral blood

**DOI:** 10.1097/MD.0000000000019325

**Published:** 2020-02-21

**Authors:** Shiho Takenaka, Norihiko Sukenaga, Masaki Ohmuraya, Yuka Matsuki, Lynn Maeda, Yumiko Takao, Munetaka Hirose

**Affiliations:** a Department of Anesthesiology and Pain Medicine; b Department of Genetics, Hyogo College of Medicine, Hyogo; c Department of Anesthesiology and Reanimatology, Faculty of Medicine Sciences, University of Fukui, Fukui; d Department of Anesthesiology and Pain Management, Nishinomiya Municipal Central Hospital, Hyogo, Japan.

**Keywords:** DN4, epigenetics, neuropathic pain

## Abstract

Elucidation of epigenetic mechanisms correlating with neuropathic pain in humans is crucial for the prevention and treatment of this treatment-resistant pain state. In the present study, associations between neuropathic pain characteristics and DNA methylation of the *transient receptor potential ankyrin 1 (TRPA1)* gene were evaluated in chronic pain patients and preoperative patients. Pain and psychological states were prospectively assessed in patients who suffered chronic pain or were scheduled for thoracic surgery. Neuropathic characteristics were assessed using the Douleur Neuropathique 4 (DN4) questionnaire. DNA methylation levels of the CpG islands in the *TRPA1* gene were examined using whole blood. Forty-eight adult patients were enrolled in this study. Increases in DNA methylation rates at CpG -51 showed positive correlations with increases in the DN4 score both in preoperative and chronic pain patients. Combined methylation rates at CpG -51 in these patients also significantly increased together with increase in DN4 scores. Neuropathic pain characteristics are likely associated with methylation rates at the promoter region of the *TRPA1* gene in human peripheral blood.

## 1 Introduction

Neuropathic pain, which is caused by a lesion or disease of the somatosensory nervous system, is a painful condition contributing to depression, anxiety, and poor quality of life. Although associations between epigenetic changes and neuropathic pain have been examined in animal models of neuropathic pain, these have not been well evaluated in humans.^[1,2]^ Elucidation of epigenetic mechanisms correlating with neuropathic pain in humans is crucial for the prevention and treatment of this treatment-resistant pain state.

Epigenetic alterations include histone modifications, DNA methylation, and non-coding RNAs.^[1]^ DNA methylation levels of CpG at -628 bp of the first exon of *transient receptor potential ankyrin 1 (TRPA1)* in whole blood was previously shown to be associated with heat or pressure pain thresholds in healthy humans.^[3–5]^ Associations between methylation rate of CpG and neuropathic pain, however, have not been evaluated, although increases in DNA methylation levels of CpG at -51 bp of *TRPA1* (GRCh37/hg19, Chr8:72987870) in whole blood have been shown to have a significant correlation with neuropathic pain characteristics in chronic pain patients.^[6]^

The Douleur Neuropathique 4 (DN4) questionnaire, which includes 10 pain characteristics, was developed to screen for neuropathic pain.^[7,8]^ These neuropathic pain characteristics reportedly correlate with pain intensity, depression, and anxiety in patients with chronic pain^[9,10]^ and cancer pain.^[11,12]^ Since neuropathic pain is 1 of 4 mechanistic descriptors for chronic pain states (nociceptive pain, neuropathic pain, nociplastic pain, and pain of unknown origin), neuropathic pain is caused by heterogenous diseases.^[13,14]^ Therefore, it would be consistent to evaluate associations between epigenetic mechanisms and neuropathic pain even in patients with or without heterogenous pain diseases.

To reveal the associations of DNA methylation of *TRPA1* with neuropathic pain and psychological variables, we examined neuropathic pain characteristics and psychological states, and measured the methylation rate at the promoter region of the *TRPA1* gene, including CpG -51 in the whole blood of patients who suffered chronic pain or were scheduled to undergo thoracic surgery for lung cancer.

## 2 Methods

This study was approved by the Ethics Committee of the Hyogo College of Medicine (#0239) and was registered in the UMIN Clinical Trials Registry (UMIN000014908).

### 2.1 Population

A total of 48 patients who were under treatment for chronic pain (n = 24), defined as pain that lasted or recurred for more than 3 months, or were scheduled for thoracic surgery for lung cancer (n = 24), were enrolled in this prospective study. Written informed consent was obtained from all participants. Eligibility criteria were age over 20 years and the American Society of Anesthesiologists physical status I–III. Exclusion criteria included presence of a psychiatric or neurologic disorder, liver or renal dysfunction, and previous thoracic surgery. All the enrolled participants completed psychological and pain assessments at the pain clinic for patients with chronic pain, or at the inpatient ward for preoperative patients at the Hyogo College of Medicine Hospital. The present study included the previous data of 12 patients with chronic pain who participated in our preliminary study,^[6]^ under the approval of the Ethics Committee of the Hyogo College of Medicine (#0239).

### 2.2 Pain assessments

Pain intensity was assessed using a numerical rating scale (NRS). The NRS, which consists of assessment using a 0 to 10 point scale, was used to assess pain intensity at rest. The lowest value (0) was labeled “no pain” and the highest value (10) was labeled “worst imaginable pain.” Pain intensity was divided into 3 grades, namely NRS = 0: no pain, NRS = 1 to 3: mild-moderate pain, and NRS ≥4: severe pain.

We used the DN4 questionnaire to discriminate neuropathic pain from other pain states.^[7,8]^ The DN4 questionnaire evaluates 10 items: characteristics of pain (burning [1], painful cold [2], electric shocks [3]), symptoms in the region of pain (tingling [4], pins and needles [5], numbness [6], itching [7]), localized pain (hypoesthesia to touch [8], hypoesthesia to pricking [9]) and pain caused or increased by brushing in the painful area (10). Items #1 to #7 of the DN4 questionnaire are answered by interviewing patients, and items #8 to #10 require examination of patients. DN4 score is a total count of these 10 items for each patient, and the cut-off value for the diagnosis of neuropathic pain is a score of 4/10.^[7,8]^ DN4 scores were graded as 3 levels, that is, DN4 of 0: no pain characteristics, DN4 of 1 to 3: non-neuropathic pain, and DN4 ≥4: neuropathic pain.

### 2.3 Psychological assessments

The self-rating questionnaire for depression (SRQ-D) was used to evaluate the state of depression,^[15]^ and the state-trait anxiety inventory 1 (STAI-1) was used to assess anxiety levels.^[16]^ SRQ-D scores were graded as 3 levels: SRQ-D ≤9: normal, SRQ-D = 10 to 15: borderline, and SRQ-D ≥16: mild depression. STAI-1 scores were graded as STAI-1 <40: low anxiety, and STAI ≥40: high anxiety levels.

### 2.4 Blood examination

To examine the DNA methylation rates at the CpG site of the *TRPA1* gene, peripheral blood was collected from each patient after conducting the interviews and physical examinations for pain and psychological assessments, and stored at −80°C until analyzed. Genome-wide assays of DNA methylation were performed using the Illumina HumanMethylation450 BeadChip (Illumina Inc., San Diego, CA) by G&G Science Co., Ltd. (Fukushima, Japan), and 6 *β*-values in the CpG-island at CpG -105, CpG -97, CpG -53, CpG -51, CpG -19, and CpG -17 (Chr8:72987924, Chr8:72987916, Chr8:72987872, Chr8:72987870, Chr8:72987838, and Chr8:72987836: GRCh37/hg19) of the *TRPA1* gene were selected in chronic pain patients. Each *β*-value represents the methylation rate of each analyzed CpG site. In preoperative patients, bisulfite next-generation sequencing (NGS) analysis was used to detect the DNA methylation rate of CpG islands from -203 to -17 at the promoter site of *TRPA1* (Takara Bio Inc. Kusatsu, Japan). Since both methods of HumanMethylation450 and NGS analysis use bisulfite, results of methylation rate correspond to each other. DNA methylation rate reportedly shows a moderate to strong correlation between 2 methods using HumanMethylation450 and bisulfite NGS analysis.^[17]^

The patients’ serum concentrations of C-reactive protein (CRP) surgery were also measured at our clinical laboratory. The normal range for CRP is below 0.3 mg.dL^−1^ in our institute.

### 2.5 Statistics

All statistical testing was 2-sided with a significance level of 5% and was performed using JMP Pro version 13.1.0 software (SAS Institute Inc. Cary, NC). We performed univariate regression analysis to investigate associations between pain states, psychological states, and the rate of DNA methylation. The Kruskal–Wallis test, followed by the Wilcoxon test or Chi-square test was used to compare patient demographics, pain, and psychological states.

## 3 Results

### 3.1 Patient demographics

Table 1 shows the demographics of the 48 patients. Among preoperative patients (n = 24), 5 patients had pain at the site of surgery before thoracic surgery for lung cancer, where pain assessments were performed. Patients with chronic pain (n = 24) had chronic low back pain (n = 16) and postherpetic neuralgia (n = 8). There were no significant differences in age (*P* = .2264) and body mass index (*P* = .9562) between preoperative and chronic pain patients. There were significant differences in sex (*P* = .0417), NRS scores representing pain intensity (*P* < .0001), DN4 scores (*P* < .0001), and SRQ-D scores for evaluating the state of depression between the 2 patient groups (*P* = .0465). STAI-1 scores for assessing anxiety levels were not significantly different between preoperative and chronic pain patients (*P* = .1762). Serum concentrations of CRP were within normal levels in all patients, and showing no significant differences between the 2 groups (Table 1).

**Table 1:**
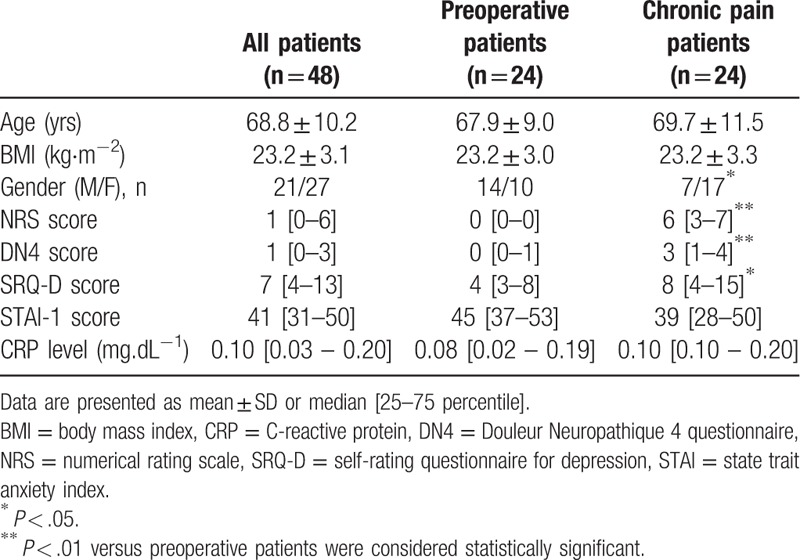
Patient demographics.

### 3.2 Neuropathic pain characteristics in preoperative patients and chronic pain patients

In preoperative patients, 1 patient (4.2%) had neuropathic pain, and 5 patients (20.8%) had non-neuropathic pain. In chronic pain patients, neuropathic pain was observed in 4 patients with postherpetic neuralgia (50.0%) and in 6 patients with chronic low back pain (37.5%). Non-neuropathic pain was observed in 3 patients with postherpetic neuralgia (37.5%), and 8 patients with chronic low back pain (50.0%).

### 3.3 Univariate analysis between DNA methylation rates, NRS, DN4, and SRQ-D scores

**Figure 1:**
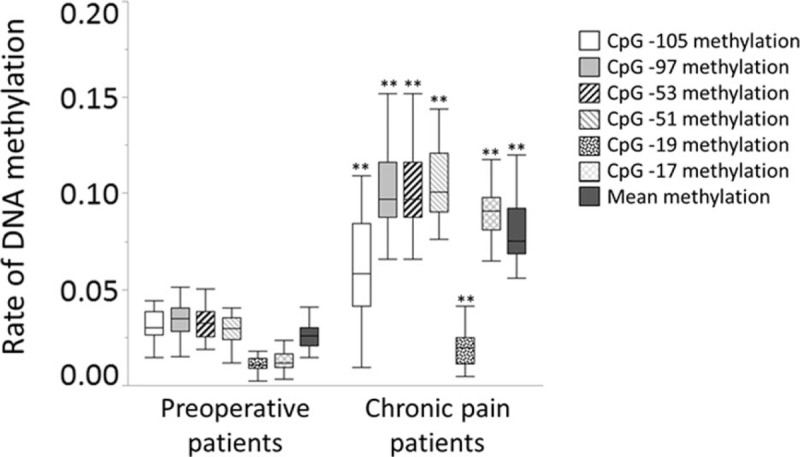
Comparison of DNA methylation rates between preoperative and chronic pain patients. ^∗∗^*P* < .01 versus preoperative patients was considered statistically significant.

There were significant differences in DNA methylation rates of CpG at -105, -97, -53, -51, -19, and -17 of the first exon of the *TRPA1* in the peripheral blood of preoperative and chronic pain patients (Fig. 1). Mean methylation rate of these 6 CpG sites also showed significant differences between preoperative and chronic pain patients (Fig. 1). DNA methylation rates at CpG -51 were positively associated with the DN4 scores both in chronic pain patients and preoperative pain patients. Combined methylation rates at CpG -51 in these patients were also significantly associated with the DN4 scores (Table 2). Furthermore, combined methylation rates at the other CpG sites examined in all patients showed positive correlations with the NRS and DN4 scores (Table 2). Combined methylation rates at CpG -105 and CpG -51 were significantly associated with SRQ-D scores (Table 3).

**Table 2:**
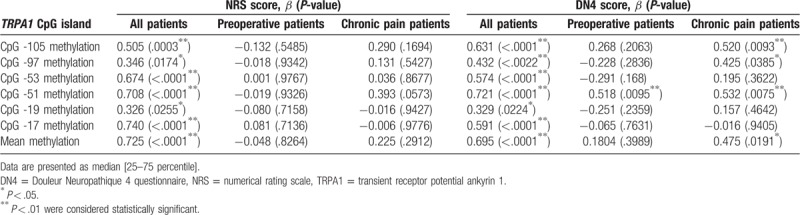
Univariate regression analyses for associations between pain states and DNA methylation levels of CpG islands at *TRPA1* gene.

**Table 3:**
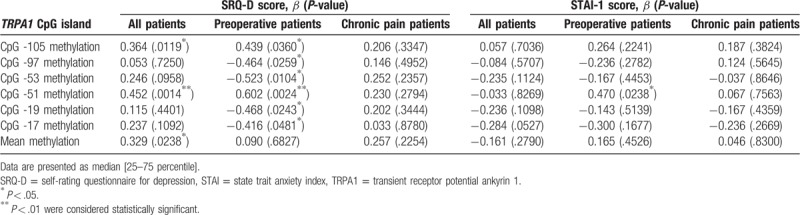
Univariate regression analyses for associations between psychological states and DNA methylation levels of CpG islands at TRPA1 gene.

### 3.4 Associations between DNA methylation rates and pain states in all patients

**Figure 2:**
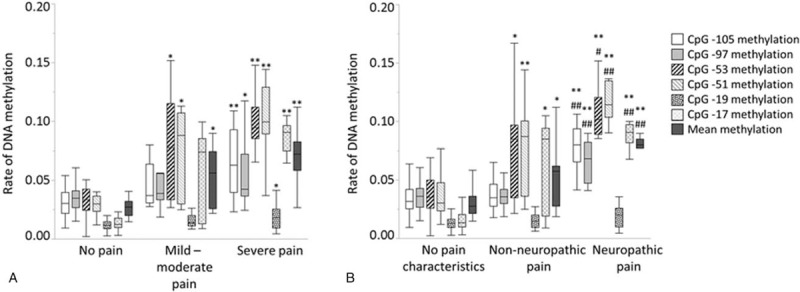
Associations between DNA methylation rates of *TRPA1* and pain intensity (A) and neuropathic pain state (B) in all patients. Differences in pain intensity with ^∗^*P* < .05 and ^∗∗^*P* < .01 versus no pain were considered statistically significant (A). Differences in neuropathic pain state with ^∗^*P* < .05 and ^∗∗^*P* < .01 versus no pain characteristics, and ^#^*P* < .05 and ^##^*P* < .01 versus nonneuropathic pain were considered statistically significant (B).

Pain intensity was graded as no pain, mild-moderate pain, and severe pain in 24, 8 and 16 patients, respectively. In patients with severe pain, all the examined CpG sites showed significant increases in combined methylation rates compared to those in patients without pain (Fig. 2A). The number of patients without neuropathic pain characteristics (DN4 score = 0) was 22, that with non-neuropathic pain (DN4 score = 1–3) was 8, and that with neuropathic pain (DN4 score ≥4) was 18. Combined DNA methylation rates at CpG -53, CpG -51, and CpG -17 significantly increased in the order of the increase in DN4 score (Fig. 2B). Mean methylation rate of the 6 CpG sites also significantly increased in the order of the increase in DN4 score (Fig. 2B).

### 3.5 Associations between DNA methylation rates and psychological states in all patients

**Figure 3:**
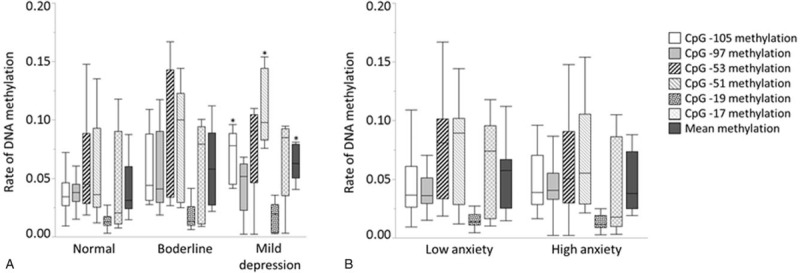
Associations between DNA methylation rate of *TRPA1* and depression (A) and anxiety (B) in all patients. Differences in depression status with ^∗^*P* < .05 versus normal were considered statistically significant (A).

Normal, borderline and mild depression states were seen in 34, 9, and 5 patients, respectively. Combined methylation rates at CpG -105 and CpG -51, and also mean methylation rate in patients with mild depression were significantly higher than in normal patients (Fig. 3A). Twenty and 28 patients, respectively showed low and high anxiety levels. There were no significant differences in methylation rates between patients with low and high anxiety levels (Fig. 3B).

## 4 Discussion

Several previous reports have stated that the level of DNA methylation, which is one of the principal mechanisms of epigenetic changes, in the peripheral blood of humans correlates with chronic pain in patients with persistent postsurgical pain,^[18–20]^ fibromyalgia,^[21]^ and chronic widespread musculoskeletal pain.^[22]^ Associations between neuropathic pain and DNA methylation, however, have not been well evaluated. Our results showed a positive correlation between neuropathic pain states and DNA methylation levels of CpG -97, -53 and -51 in the promoter region of *TRPA1*in the whole blood, which corresponds to the relationship between the DN4 score and DNA methylation rate at CpG -51 of *TRPA1* in chronic pain patients shown in our previous study.^[6]^ These results suggest that DNA methylation at the promoter region of *TRPA1* in peripheral blood might be associated with the presence of neuropathic pain characteristics in humans.

Over the half of chronic pain patients had neuropathic pain in the present study. The number of neuropathic pain characteristics are also reportedly high in patients with postherpetic neuralgia^[23,24]^ or chronic low back pain.^[25,26]^ On the other hand, few patients showed neuropathic pain in preoperative patients in the present study. Even in preoperative patients whose DN4 score and DNA methylation rate were relatively low compared to chronic pain patients, the methylation rate at CpG -51 was significantly associated with the DN4 score in preoperative patients, same as in the results of chronic pain patients. Given that the significant relationship between DN4 scores and DNA methylation rates at CpG -51 of *TRPA1* shown in our preliminary study,^[6]^ this site might be predominantly associated with neuropathic pain characteristics.

Gombert et al^[4]^ reported that an increase in CpG -628 methylation significantly correlates with a decrease in the pressure pain threshold in healthy subjects. The methylation rate at CpG -51, however, showed no correlation with pressure pain threshold.^[4]^ Since a low pressure pain threshold is observed in patients with neuropathic pain,^[27,28]^ further investigations are needed to evaluate the association between pressure pain threshold and DNA methylation at CpG -51 in patients under treatment for neuropathic pain.

Increases in the methylation rates of *TRPA1* tended to be associated with pain intensity or depression, but not with anxiety, in the present study. Although neuropathic pain characteristics correlate with pain intensity and depression,^[9–12]^ there might be no clear relationship between *TRPA1* methylation rates, pain intensity and depression. Therefore, DNA methylation of the *TRPA1* is unlikely a confounder for pain intensity or depression in neuropathic pain states.

Sensitivity of the diagnosis of neuropathic pain using the Japanese DN4 questionnaire was 71%, which was lower than specificity of 92% in adult patients with heterogenous diseases.^[8]^ Therefore, the DN4 questionnaire results in few patients who have no neuropathic pain being told of the possibility that they have neuropathic pain.^[29]^ We suggest that prevalence of neuropathic pain was likely underestimated in the present study.

A limitation of this study is that the origin of the *TRPA1* gene in whole blood is unclear. TRPA1 expression as a nociceptor at primary sensory neurons plays pivotal roles in the development and maintenance of neuropathic pain.^[30,31]^ On the other hand, TRPA1 expressed in immune cells also contributes to chronic pain.^[32]^ Although methylation rates of the *TRPA1* promoter region can be a biomarker for neuropathic pain, the origin of the *TRPA1* gene in peripheral blood do not necessarily correlate with the peripheral nervous system and immune cells.^[33,34]^ Further investigations are needed to reveal the origin of the *TRPA1* gene.

Another limitation of this study is that the effects of drugs used for neuropathic pain on methylation rates of the *TRPA1* gene cannot be excluded. Following to the guidelines for the pharmacological management of neuropathic pain,^[35]^ anticonvulsants, antidepressants, or opioids were prescribed for treatment of neuropathic pain in the present study. There is a growing body of evidence suggesting that the significance of DNA methylation in drug dependence.^[36]^ Further investigations are required to elucidate whether DNA methylation of *TRPA1* is directly associated with mechanisms of neuropathic pain or is caused by other effects including treatments.

## 5 Conclusion

DNA methylation rates at CpG islands of the promoter region of *TRPA1* are likely associated with neuropathic pain characteristics in humans.

## Author contributions

**Conceptualization:** Munetaka Hirose.

**Data curation:** Shiho Takenaka, Norihiko Sukenaga.

**Investigation:** Shiho Takenaka, Norihiko Sukenaga.

**Methodology:** Munetaka Hirose, Masaki Ohmuraya.

**Project administration:** Munetaka Hirose.

**Supervision:** Munetaka Hirose Masaki Ohmuraya, Yumiko Takao.

**Validation:** Masaki Ohmuraya, Yuka Matsuki, Lynn Maeda, Yumiko Takao

**Writing – original draft:** Munetaka Hirose, Shiho Takenaka, Masaki Ohmuraya.

**Writing – review and editing:** Munetaka Hirose.

Munetaka Hirose orcid: 0000-0003-1291-2827.
